# Morphological and Molecular Evidence for a New Species of *Ilex* (Aquifoliaceae) from Guangdong, China, with Insights into Its Phylogenetic Position Within *Ilex* sect. *Ilex*

**DOI:** 10.3390/plants15101431

**Published:** 2026-05-08

**Authors:** Yizhe Zhao, Xiaosa Huang, Lei Jiang, Peng Zhou, Zhiyi Xie, Qiang Fan, Kewang Xu

**Affiliations:** 1 Co-Innovation Center for Sustainable Forestry in Southern China, Key Laboratory of National Forestry and Grassland Administration on Subtropical Forest Biodiversity Conservation, College of Life Science, Nanjing Forestry University, Nanjing 210037, China; 2 Guangzhou Linfang Ecology Co., Ltd., Guangzhou 510650, China; 3 Botanical-Perception Studio, Nangiing 210046, China; 4 Jiangsu Academy of Forestry, Nanjing 211153, China; 5 Guangdong Ecological and Environmental Monitoring Center, Guangzhou 510308, China; 6 State Key Laboratory of Biocontrol, Guangdong Provincial Key Laboratory of Plant Resources, School of Life Sciences, Sun Yat-Sen University, Guangzhou 510275, China

**Keywords:** biodiversity, conservation, molecular phylogeny, taxonomy

## Abstract

*Ilex lanceifolia* K.W.Xu & Lei Jiang, a new species from the western Pearl River Delta of Guangdong, China, is described based on morphological and molecular evidence. To test whether this newly discovered population represents a distinct lineage and to assess the congruence between leaf morphology and phylogeny, we integrated multivariate morphometrics, scanning electron microscopy, and phylogenetic analyses of nuclear ITS, ETS, and nepGS sequences. The new species resembles *I. xiaojinensis* and *I. peiradena* in shrubby habit and lanceolate leaves but differs by its prominently raised abaxial leaf veins forming distinct reticulate areoles, and green to purplish-black petioles and young branchlets. However, phylogenetic analyses unexpectedly place it within *Ilex* sect. *Ilex* forming a clade with *I. graciliflora* and six other species, rather than with its morphological look-alikes. This discordance strongly suggests that the lanceolate leaf shape has evolved convergently in multiple lineages of *Ilex*, likely as an adaptive strategy to the high-humidity, low-light understory conditions of subtropical lowland forests. The new species is currently known only from a single population in Jiangmen City, with several thousand individuals but an extremely restricted range (<20 km^2^), warranting conservation attention. This discovery highlights the underestimated biodiversity of lowland forests in the Pearl River Delta and underscores the need to prioritize remnant habitat fragments in rapidly urbanizing regions.

## 1. Introduction

The genus *Ilex* L. (Aquifoliaceae), comprising over 600 species of dioecious trees and shrubs, is the sole extant genus within its family [1,2,3]. It exhibits a sub-cosmopolitan but highly uneven distribution, with major centers of diversity in East Asia and tropical America, and fewer representatives in North America, Europe, Africa, Australia, and various oceanic islands [1,4]. China represents one of the primary diversity hotspots for the genus, harboring more than 210 species [5,6]. These species are distributed across the southern slopes of the Qinling Mountains, the Yangtze River basin, and the vast regions to the south, with the highest species richness concentrated in southwestern China (e.g., Yunnan, Sichuan, Guizhou) and southern China (e.g., Guangxi, Guangdong) [7]. More than half of these species are endemic to China, and a substantial proportion have narrow distributions, often restricted to specific mountain ranges or isolated habitats [6]. As a result, with the continuous advancement of biodiversity surveys in recent years, numerous new species with narrow distributions have been discovered and described [5,6,8,9,10,11].

Historically, the classifications of *Ilex* have relied heavily on morphological characters [12,13,14]. Molecular phylogenetics has since revealed extensive incongruence between plastid and nuclear DNA, attributed to frequent hybridization and reticulate evolution [3,15,16]. Recent work by Yang et al. [2], integrating nuclear markers with morphology and distribution, has advanced our understanding by proposing a sectional classification that better reflects monophyletic lineages. Among the recognized infrageneric groups, *Ilex* sect. *Ilex* (syn. sect. *Aquifolium*) represents one of the most diversified and geographically widespread lineages within the genus *Ilex*. Distributed primarily across temperate to subtropical Eurasia, this section is characterized by evergreen habit, leaves that are often spiny or serrate, compact pseudoracemes or pseudopaniculate infructescences, and drupes typically containing four palmately striate and sulcate pyrenes [2].

**Figure 1:**
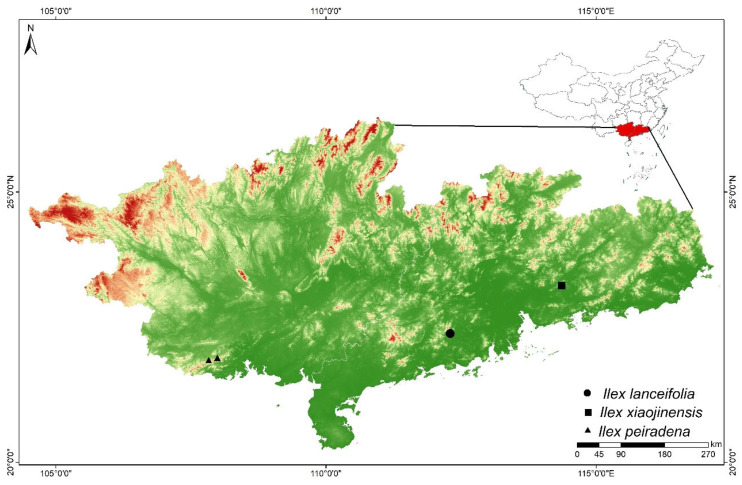
Distribution map of *Ilex lanceifolia* sp. nov. and its morphologically similar species in Guangdong and Guangxi, China.

During recent botanical explorations in Xikeng Forest Farm, Jiangmen City, Guangdong Province (Figure 1), a distinct and previously undocumented species of *Ilex* was discovered. This species exhibits a unique combination of morphological traits, including a small shrubby habit, lanceolate leaves with subentire or minutely glandular-serrate margins, leaf veins prominently raised on the abaxial surface with veinlets anastomosing into distinct reticulate areoles, green to purplish-black petioles and young branchlets, fascicled axillary inflorescences, and 4-merous flowers with longitudinally grooved mature fruits, that distinguishes it from all known species within *Ilex*. sect. *Ilex*. Notably, this is the second new species of *Ilex* recently discovered in the Jiangmen area, following the discovery of *Ilex. Jiangmenensis* Lei Jiang & K.W. Xu [10]. Here, we describe it as a new species, *Ilex lanceifolia*, providing detailed morphological characterization, comparative analyses with phenotypically similar species, and a discussion of its phylogenetic placement and conservation status.

In this study, we address two primary questions: (1) Does the newly discovered population in Guangdong represent a distinct lineage warranting species status? (2) To what extent does leaf morphology reflect phylogenetic relationships in this section? We hypothesize that (i) *I. lanceifolia* is a distinct species separable from its morphologically similar congeners by a combination of quantitative and qualitative leaf traits, and (ii) the lanceolate leaf shape has evolved convergently in multiple *Ilex* lineages, leading to discordance between morphological similarity and phylogenetic relatedness.

## 2. Results

### 2.1. Morphological Characteristics and Comparison with Similar Species

**Figure 2:**
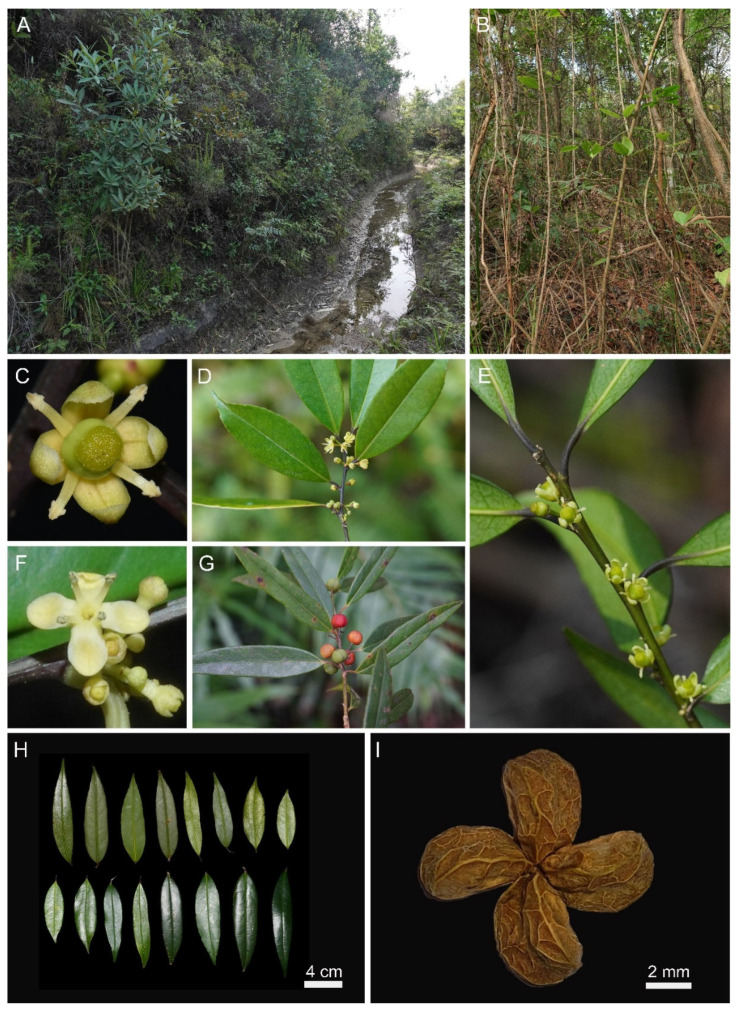
Habitat and morphological characters of *Ilex lanceifolia* sp. nov. (**A**,**B**) Habitat. (**C**) Female flower. (**D**) Male inflorescence and flowers. (**E**) Female inflorescence and flowers. (**F**) Male flower. (**G**) Infructescences and fruits. (**H**) Leaf morphology (scale = 4 cm). (**I**) Pyrenes (scale = 2 mm).

**Figure 3:**
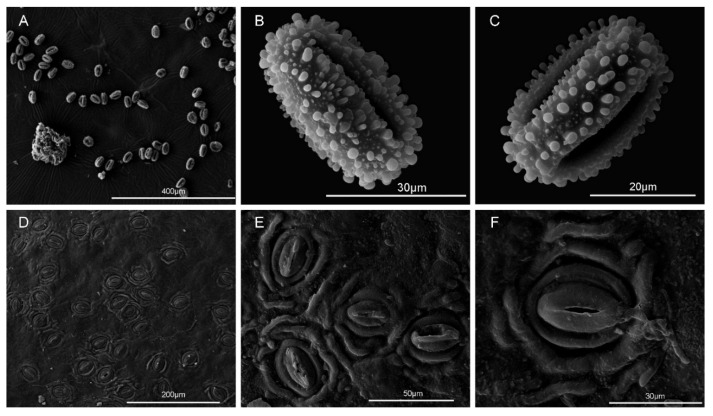
Scanning electron microscopy (SEM) of *Ilex lanceifolia* sp. nov. (**A**–**C**) Pollen grains. (**D**–**F**). Stomata.

The new species is an evergreen shrub with lanceolate leaves, 4-merous yellow flowers, and globose red fruits bearing four pyrenes (Figure 2). The pollen grains are tricolporate, prolate, with a polar axis of ca. 38.32 μm and an equatorial axis of ca. 25.28 μm; the exine ornamentation consists of conspicuous coarse verrucae densely distributed over the entire surface, with rounded verrucae ca. 1–2 μm in diameter (Figure 3A–C). Stomata are cyclocytic, confined to the abaxial leaf surface; average stomatal size is 34.2 × 24.5 μm (Figure 3D–F).

**Figure 4:**
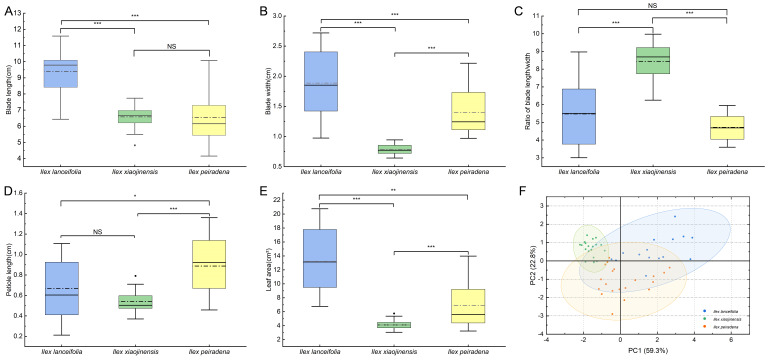
Comparison of leaf morphological characters among *Ilex lanceifolia* sp. nov. and its morphologically similar species *I. xiaojinensis*, and *I. peiradena*. (**A**–**E**) Boxplots showing leaf length, leaf width, length/width ratio, petiole length, and leaf area of the three species. The box plots show the median (solid horizontal line), mean (dash-dotted line), interquartile range (box), and range (whiskers). Significant differences between species are indicated by asterisks: * *p* < 0.05, ** *p* < 0.01, *** *p* < 0.001; NS, not significant. (**F**) Principal component analysis (PCA) of leaf morphological traits based on the same five variables, showing segregation of the three species along the first two principal components, which together account for 82.04% of the total variance (PC1: 59.28%; PC2: 22.76%). In the PCA plot, ellipses represent 95% confidence intervals for each species. Statistical validation of this segregation is provided by MANOVA (Wilks’ Lambda = 0.086, F_10,96_ = 23.22, *p* < 0.001; see Section 3). Sample sizes: *n* = 15 for *I. lanceifolia*; *n* = 20 for *I. xiaojinensis*; *n* = 20 for *I. peiradena*.

A one-way MANOVA on five quantitative leaf traits (55 individuals: 15 *Ilex lanceifolia*, 20 *Ilex xiaojinensis*, 20 *Ilex peiradena*) revealed a highly significant species effect (Wilks’ Lambda = 0.086, F_10,96_ = 23.22, *p* < 0.001), confirming that the three species are morphologically distinct. This statistical validation supports the PCA clustering (Figure 4F), where the first two principal components captured 82.04% of the total variance (PC1: 59.28%; PC2: 22.76%). Morphometric comparisons (Figure 4) show that *I. lanceifolia* bears the longest leaf blade and largest leaf area among the three species, while its petiole length and leaf width are comparable to those of the other two. Bonferroni post hoc tests further identify a key difference in leaf shape: the leaf length/width ratio of *I. lanceifolia* is significantly lower than that of *I. xiaojinensis* (*p* < 0.001) but not significantly different from that of *I. peiradena* (*p* = 0.185). Collectively, the morphometric evidence strongly supports recognizing *I. lanceifolia* as a distinct species.

### 2.2. Phylogenetic Position of the New Species

**Figure 5:**
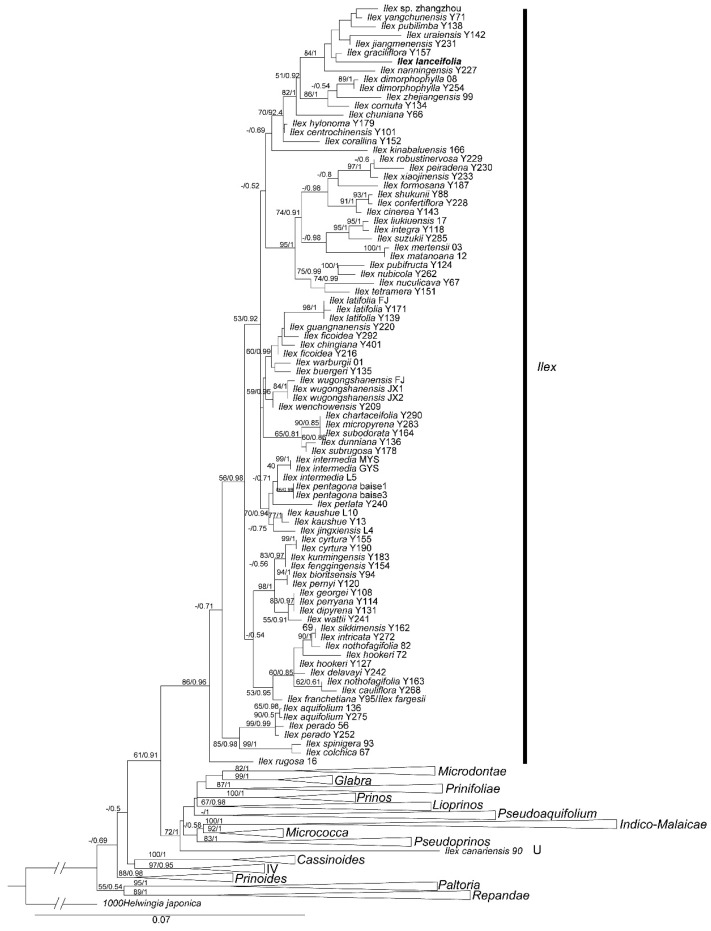
Maximum likelihood (ML) phylogeny of *Ilex* inferred from combined ITS, ETS, and nepGS sequence data. The phylogeny primarily focuses on the relationships within *Ilex.* sect. *Ilex*, with other sections collapsed into simplified clades for clarity. Numbers near each branch are maximum-likelihood bootstrap support (MLBS) and Bayesian inference posterior probability (BIPP). Dash means that MLBS ≤ 50% or BIPP ≤ 0.5. The new species *Ilex lanceifolia* sp. nov. is indicated in bold in the ML tree.

The aligned nuclear dataset comprised 2439 bp, of which 1368 sites were constant, 670 were parsimony-informative, and 401 were variable but parsimony-uninformative. Based on the Akaike information criterion [17], ModelFinder selected GTR + F + I + G4 as the best-fit nucleotide substitution model for both maximum likelihood (ML) and Bayesian inference (BI) analyses. When the concatenated dataset was used, the tree topologies obtained from ML and BI analyses were generally congruent. The ML phylogeny of the genus *Ilex* and outgroups, inferred from the concatenated data, is presented in Figure 5.

Consistent with our morphological predictions, the new species was recovered as a member of *Ilex.* sect. *Ilex* in the phylogenetic tree. Together with *I. graciliflora* Champ., *I. nanningensis* Hand.-Mazz., *I. jiangmenensis*, *I. pubilimba* Merr. & Chun, *I. uraiensis* Mori & Yamam., *I. yangchunensis* C. J. Tseng, and a species (*I*. sp.) from Fujian, China, it formed a strongly supported clade (MLBS: 84%; BIPP: 1). This clade was moderately resolved as sister to a clade comprising *I. cornuta* Lindl. & Paxton, *I. dimorphophylla* Koidz., and *I. zhejiangensis* C. J. Tseng ex S. K. Chen & Y. X. Feng. Within this clade, the new species forms a weakly supported sister relationship with *I. graciliflora* in the phylogenetic tree, but not with its morphologically most similar species *I. xiaojinensis* and *I. peiradena*.

## 3. Discussion

### 3.1. Morphological Comparison

*Ilex lanceifolia* is an evergreen species with serrate leaf margins, inflorescences as fasciculate cymes borne in leaf axils, and drupes typically containing four palmately striate and sulcate pyrenes. These characteristics firmly place it as a member of *Ilex.* sect. *Ilex* [2]. Within this section, evergreen shrubs with narrowly lanceolate leaves include *I. xiaojinensis* and *I. peiradena* [7]. Morphologically, *I. lanceifolia* most closely resembles *I. xiaojinensis* (both are small shrubs with slender branchlets, narrowly lanceolate leaves, obtusely serrulate margins, fasciculate cymes, and red fruits longitudinally 8-sulcate), but differs by having larger leaf blades (8.3–11.6 cm vs. 5.5–7.7 cm in length; 1.0–2.4 cm vs. 0.7–0.9 cm in width), longer petioles (0.4–1.1 cm vs. 0.35–0.8 cm), greater leaf area (9.3–20.7 cm^2^ vs. 3.0–5.8 cm^2^), a smaller length/width ratio (3.0–6.9 vs. 7.6–10.0; Figure 4A–E). Compared to *I. peiradena*, the new species can be distinguished by its larger leaf blades (8.3–11.6 cm vs. 4.1–10.0 cm in length; 1.4–2.4 cm vs. 0.95–2.2 cm in width), longer petioles (0.4–1.1 cm vs. 0.45–1.35 cm), larger leaf area (9.3–20.7 cm^2^ vs. 3.2–13.8 cm^2^), and smaller length/width ratio (3.0–6.9 vs. 3.6–5.9; Figure 4A–E). Principal component analysis (PCA) based on morphological characters shows that *I. lanceifolia* is clearly separated from *I. xiaojinensis* and *I. peiradena* along the first two principal components, which explain 59.3% and 22.8% of the total variance, respectively (Figure 4F).

While individual morphological traits can sometimes exhibit overlap due to ecological plasticity, our multivariate analysis (MANOVA) confirms that the combined character suite of *I. lanceifolia* is statistically distinct (Wilks’ Lambda = 0.086, *p* < 0.001). The Principal Component Analysis (PCA) further demonstrates that *I. lanceifolia* forms a discrete, non-overlapping cluster in the morphometric space, separated from *I. xiaojinensis* and *I. peiradena* along the first two principal components. The lack of morphological transition between these clusters, despite their occurrence in similar subtropical habitats, suggests that these diagnostic features are genetically fixed rather than transient plastic responses to environmental factors. This clear statistical and visual separation (Figure 4F) provides strong evidence that *I. lanceifolia* is a distinct species.

### 3.2. Phylogenetic Placement of the New Species

The phylogenetic position of the new species was further determined by conducting ML and BI analyses based on three nuclear sequences. With *Helwingia* as the outgroup, the genus *Ilex* was recovered as a well-supported monophyletic group (Figure 5), consistent with previous phylogenetic studies of Aquifoliaceae [2,3,5]. Using a similar data matrix, our phylogenetic tree exhibited a topology largely congruent with that reported by Yang et al. [2]. All sampled *Ilex* accessions were divided into 16 major clades, delimited by a combination of morphological and distributional characters (Figure 5) [2]. No well-supported conflicts were detected between our tree topologies and those of Yang et al. [2]. However, the deep nodes within the genus showed some discrepancies between the two analyses, mainly due to the low resolution of phylogenetic trees reconstructed from the limited nuclear markers (Figure 5). These inconsistencies suggest that the current backbone phylogeny of *Ilex* remains unstable when based solely on limited nuclear markers. Future phylogenetic studies employing more comprehensive datasets are urgently needed for this taxonomically challenging genus [3].

Within our phylogenetic tree, *Ilex lanceifolia* formed a strongly supported clade with *I. graciliflora*, *I. nanningensis*, *I. jiangmenensis*, *I. pubilimba*, *I. uraiensis*, *I. yangchunensis*, and an unidentified species from Fujian, indicating a close phylogenetic relationship among these taxa (Figure 5). This clade belongs to *Ilex.* sect. *Ilex* as circumscribed by Yang et al. [2]. in their phylogenetic tree. In fact, these species were initially assigned to *Ilex.* sect. *Ilex* (sect. *Aquifolium*) based on morphological evidence, including being evergreen trees or shrubs, with inflorescences fascicled and borne in leaf axils of two-year-old or even older branches, and having four (rarely fewer) pyrenes per fruit, which are striate (or ridged) and sulcate, or variously wrinkled and foveolate [18]. Recently, Yang et al. [2] also confirmed their placement in *Ilex.* sect. *Ilex* using phylogenetic analyses. Therefore, our molecular results unequivocally place the new species within *Ilex.* sect. *Ilex* (Figure 5).

Within *Ilex.* sect. *Ilex*, the new species is most closely related to *I. graciliflora* in our phylogenetic tree (Figure 5), with which it shares morphological similarities, such as inflorescences fascicled in leaf axils, four pyrenes per fruit, and palmately striate pyrenes. Nevertheless, the new species can be readily distinguished from *I. graciliflora* by being an evergreen shrub with narrowly lanceolate leaves. Morphologically, *I. lanceifolia* exhibits some vegetative resemblance to *I. xiaojinensis* and *I. peiradena* (e.g., small shrubby habit, lanceolate leaves, and four pyrenes). However, our phylogenetic analyses demonstrate that they are not closely related. Instead, *I. xiaojinensis*, *I. peiradena*, and *I*. *robustinervosa* C. J. Tseng ex S. K. Chen & Y. X. Feng form a strongly supported sister clade (MLBS: 97%; BIPP: 1) within *Ilex.* sect. *Ilex*, separated from the new species by multiple nodes (Figure 5).

One of the most interesting findings of this study is the discordance between morphological similarity and phylogenetic relatedness in *I. lanceifolia*. Although the new species exhibits a striking vegetative resemblance to *I. xiaojinensis* and *I. peiradena*, our molecular data place it in a different clade, as a weakly supported sister to *I. graciliflora*, a species that differs markedly in leaf morphology. This pattern suggests that the lanceolate leaf shape and associated vegetative traits may have evolved convergently in multiple lineages in response to similar ecological pressures. Specifically, the narrow-leaved morphotype of *I. lanceifolia* likely represents an adaptive strategy for the high-humidity and low-light understory environments of lowland subtropical forests in the Pearl River Delta. In such niches, lanceolate leaves facilitate efficient water drainage (reducing epiphyte growth) and maximize light interception. This adaptive convergence explains why *I. lanceifolia* mimics distantly related taxa like *I. peiradena* or *I. xiaojinensis*. Therefore, our results add to the growing body of evidence that morphological characters alone can be misleading in inferring phylogenetic relationships within *Ilex*. The weakly supported nodes within *Ilex.* sect. *Ilex* (e.g., the *I. lanceifolia–I. graciliflora* sister relationship) likely reflect both limited phylogenetic signal from our three-marker dataset (2439 bp with only 670 parsimony-informative sites) and underlying biological processes. Previous phylogenomic studies have demonstrated extensive cytonuclear discordance in *Ilex* [3], suggesting that incomplete lineage sorting (ILS) and historical hybridization are common in the genus. Both processes can generate weakly supported or conflicting topologies when only a few markers are used, and our data cannot distinguish between them. Future studies employing high-throughput sequencing approaches, such as target capture or whole-genome resequencing, will be required to resolve the backbone phylogeny of *Ilex.* sect. *Ilex* and to clarify the evolutionary relationships of morphologically distinctive species such as *I. lanceifolia*.

### 3.3. Distribution Ranges of Ilex lanceifolia and Morphologically Similar Species

*Ilex lanceifolia* is currently known only from a single population in Xikeng Forest Farm, Jiangmen City, Guangdong Province, China. Its distribution may be restricted to the low-elevation hills of the western Pearl River Delta region (Figure 1). In contrast, its morphologically similar species *I. xiaojinensis* is sporadically distributed in Xiaojin, Boluo County, Huizhou City, eastern Guangdong Province, approximately 150 km east-northeast of the type locality of *I. lanceifolia*. Another morphologically similar species, *I. peiradena*, is restricted to Shangsi County, Fangchenggang City, southwestern Guangxi Zhuang Autonomous Region, approximately 400 km west-southwest of the type locality of *I. lanceifolia* (Figure 1).

The highly restricted distribution of *I. lanceifolia* (single population, <20 km^2^) contrasts sharply with the broader ranges of some of its relatives, such as *I. graciliflora*, which is known from multiple localities in South China. This pattern of narrow endemism is not uncommon in *Ilex*; nearly 50% of Chinese *Ilex* species are narrow endemics, and several others, including *I. calcicola* [11], *I. danxiaensis* [5], and *I. jiangmenensis* [10] are also confined to single or a few localities with specialized habitats. The concentration of these narrow endemics in southern China, particularly in low-elevation areas that have experienced intense anthropogenic modification, suggests that many more such species may have gone extinct before being documented. Therefore, the discovery of *I. lanceifolia* not only adds to the species inventory of *Ilex* but also serves as a reminder of the conservation value of even small, isolated forest fragments in the Pearl River Delta.

### 3.4. Conservation Significance

Despite being one of the most economically developed and densely urbanized regions in China, the Pearl River Delta still harbors remnant patches of lowland evergreen broad-leaved forests. Compared to the well-explored mountain ranges of northern and western Guangdong, these lowland habitats have received relatively little botanical attention. Recent field surveys, however, have revealed several narrowly endemic plant species from these lowland habitats, including *I. jiangmenensis* [10] and several other undescribed taxa, suggesting that the biodiversity of this region remains significantly underestimated. The discovery of yet another new *Ilex* species from the same area further supports this notion and highlights the urgent need for comprehensive floristic inventories in the remaining natural habitats of the Pearl River Delta.

*Ilex lanceifolia* is currently known only from a single population in Xikeng Forest Farm, Jiangmen City. Although an estimated several thousand mature individuals have been found, its distribution range is extremely narrow, with an extent of occurrence estimated to be less than 20 km^2^. The proximity of its habitat to ongoing urban development poses specific potential threats: (1) habitat degradation from nearby agricultural expansion, (2) infrastructure construction (e.g., road building and tourism facility development), and (3) edge effects leading to microclimate changes and increased invasive species pressure. The restricted range and presence of plausible threats indicate that conservation attention is warranted. Further field surveys in adjacent areas and long-term population monitoring are needed to inform any future conservation assessment.

### 3.5. Taxonomic Treatment

***Ilex lanceifolia*** K.W. Xu & Lei Jiang, sp. nov. (Figure 2, Figure 3C and Figure 4A).

**Type.** China. Guangdong: Jiangmen City, Enping, Niujiang Town, Xikeng Forest Farm, elev. 307 m, 22°22′53″ N, 112°18′10″ E, 3 August 2024, Huang & Xu 418 (holotype SYS!; isotype NF!).

**Diagnosis.** *Ilex lanceifolia* is morphologically similar to *I. xiaojinensis* and *I. peiradena* in sharing a small shrubby habit, slender branchlets, narrowly lanceolate leaves with subentire or minutely serrate margins, fasciculate cymes borne in leaf axils, and drupes typically containing four pyrenes. However, the new species can be readily distinguished from both by its larger leaf blades and longer petioles, smaller leaf length-to-width ratio and green to purplish-black petioles and young branchlets, and leaf veins prominently raised on the abaxial surface with veinlets anastomosing into distinct reticulate areoles.

**Description.** Shrubs evergreen, dioecious, up to 3 m tall; trunk diameter up to 3 cm. Branchlets slender, terete, straight, angled when young, green to purplish-black; older branchlets terete, not angled, grayish-white, glabrous. Terminal buds small, narrowly conical, glabrous. Leaves persistent on one- to three-year-old branches. Stipules deciduous, minute; petiole abaxially terete, adaxially sulcate, (3–)5–10 mm, glabrescent; leaf blade abaxially pale yellowish-green, adaxially dark green and glossy, lanceolate or narrowly lanceolate, (6.5–)8.5–10(–11.5) × 1.5–2.5 cm, papery to thinly leathery; midvein raised abaxially, impressed and glabrescent adaxially; lateral veins (5–)6–8(–10) pairs, slightly raised on both surfaces, anastomosing near margin; reticulate veins distinct on both surfaces; base cuneate; margin crenate, with (5–)8–12(–15) inconspicuous teeth on each side, slightly recurved; apex acuminate to caudate-acuminate. Inflorescences fasciculate cymes, axillary on branches, glabrous. Male inflorescences 1–3-flowered, fascicled; pedicels 2–4 mm, with 2 basal bracteoles, deltoid; flowers 4-merous, yellowish-white; calyx patelliform, ca. 3 mm in diam., lobes 4, ovate-deltoid, ca. 1.5 mm wide at base, ca. 1 mm long; petals 4, oblong-ovate, 3–4 × ca. 2 mm, apex rounded; stamens 4, nearly as long as petals, anthers ovoid. Female inflorescences 1–3-flowered, fascicled; pedicels 2–4 mm, with 2 basal bracteoles, deltoid; flowers 4-merous, yellowish-white; calyx and corolla similar to those of male flowers; staminodes 4, ca. 3.5 mm long, anthers sterile; ovary ovoid, ca. 3.5 mm, 4-locular; style short; stigma discoid, 4-lobed. Infructescences fasciculate cymes; fruiting pedicels 3–5 mm, glabrous. Fruit globose or subglobose, 5–5.5 mm in diam., red when mature; pyrenes 4, ellipsoidal, ca. 5 mm long, abaxially palmately striate and one-sulcate; endocarp woody. Flowering occurs in March; fruiting from March to October.

**Phenology.** Flowering occurs in March, and the fruiting period extends from March to October.

**Distribution and habitat.** *Ilex lanceifolia* is known only from the type locality, Xikeng Forest Farm in Jiangmen City, Guangdong Province, China (Figure 3). It usually occurs in subtropical evergreen broad-leaved forests and forest margins at elevations of 100–400 m in the low-elevation hills of the western Pearl River Delta region.

**Conservation assessment.***Ilex lanceifolia* is endemic to Xikeng Forest Farm of Jiangmen City. Currently, only one large population with an estimated several thousand mature individuals has been found, but the distribution range is extremely narrow (extent of occurrence estimated < 20 km^2^). According to IUCN Red List criteria D2, this species should be listed as Vulnerable (VU). More extensive fieldwork in nearby mountains of the western Pearl River Delta region will be needed to accurately assess its conservation status.

**Etymology.** The specific epithet is derived from the Latin lancea (lance) and folium (leaf), referring to the narrowly lanceolate leaves of the species.

**Vernacular name.** We propose the Chinese name pīzhēnyè dōngqīng (披针叶冬青), which reflects the lanceolate leaf shape of the new species.

**Additional specimens examined (paratypes).** China. Guangdong: Jiangmen City, Enping, Niujiang Town, Xikeng Forest Farm, elev. 350 m, 22°22′56″ N, 112°18′30″ E, 6 March 2026, Huang & Xu 861 (SYS!, NF!); the same locality, Huang & Xu 862 (SYS!, NF!).

## 4. Material and Methods

### 4.1. Morphological Study

Field investigations were conducted in Jiangmen, Guangdong Province, to document the habitat, population characteristics, tree height, and phenology of the new species. Voucher specimens, including both male and female flowers as well as fruits, were collected and prepared following standard herbarium procedures. Voucher specimens were deposited in the Herbarium of Sun Yat-Sen University (SYS) and Nanjing Forestry University (NF). Morphological characters were observed and measured directly or using a stereomicroscope based on fresh and dried specimens. For pollen morphology, healthy and mature pollen grains were selected, air-dried, mounted on stubs, sputter-coated with gold, and then examined and photographed using an ESEM-Quanta 200 (FEI, Hillsboro, OR, USA). To determine pyrene morphology, mature red fruits were collected, the sarcocarp was rinsed off with distilled water, and the number of pyrenes per fruit was counted. Cleaned pyrenes were photographed using a Nikon camera. Quantitative morphological measurements were performed using ImageJ software (Version 1.54b, National Institutes of Health, Bethesda, MD, USA) based on specimens with a scale bar. In addition, morphological observations and measurements of morphologically similar species were carried out using online digital images of herbarium specimens accessed through the Chinese Virtual Herbarium (CVH, https://www.cvh.ac.cn, accessed on 6 May 2026) and our own collected specimens.

This study employed principal component analysis (PCA) to quantitatively evaluate morphological variation among the three species, based on five continuous traits (blade length, blade width, blade length/width ratio, petiole length, and leaf area) using Origin 2026 (OriginLab Corporation, Northampton, MA, USA). Prior to the analysis, all morphometric data were standardized to a mean of zero and unit variance to eliminate scale effects. The statistical significance of the observed morphological segregation patterns presented in Figure 4 was evaluated using a one-way multivariate analysis of variance (MANOVA). Wilks’ Lambda statistic was used to assess the overall effect of species on the combined suite of morphological traits. Following a significant MANOVA result, post hoc pairwise comparisons with Bonferroni correction were conducted to identify specific trait differences between *Ilex lanceifolia* and its morphologically similar congeners with similar morphology. The significance level for all statistical tests was set at *α* = 0.05.

### 4.2. Phylogenetic Study

To determine the phylogenetic position of the new species, we included a total of 235 accessions representing 192 species in our phylogenetic analyses, comprising one accessions of the new species, 234 accessions of other *Ilex* species, and one accession of outgroup species (*Helwingia japonica* (Thunb.) F. Dietr. Supplementary Materials). Based on morphological evidence observed in the field, we preliminarily assigned the new species to *Ilex*. sect. *Ilex*. Accordingly, our sampling strategy aimed to include as many members of *Ilex*. sect. *Ilex* as possible, along with representative species from each section recognized by Yang et al. Most sequences were downloaded from GenBank, while the ITS and nepGS sequences of the new species were newly generated in this study.

Genomic DNA was extracted from silica-gel-dried leaves using a modified 2 × CTAB procedure following Doyle & Doyle [19] and used as the template for polymerase chain reaction (PCR). Two nuclear markers were selected for amplification. The ITS region was amplified using primers ITSF and ITSR [6], and the nepGS fragment was amplified using primers GScp687f and GScp994r [20]. Each PCR was carried out in a 50 µL mixture containing 30 µL of 2 × PCR Master Mix (containing 2 × Taq DNA polymerase, 2 × PCR buffer, 2 × dNTP, and Mg^2+^; Tiangen), 14 µL of ddH_2_O, 1 µL of each primer, and 4 µL of DNA template. The thermocycling conditions for both ITS and nepGS consisted of an initial denaturation at 95 °C for 4 min, followed by 35 cycles of 95 °C for 45 s, 58 °C for 1 min, and 72 °C for 2 min, with a final extension at 72 °C for 10 min. PCR products were purified and sequenced by Sangon Biological Technology (Shanghai, China).

The newly generated raw sequences were assembled and edited using Sequencher (Version 4.14, Ann Arbor, MI, USA). All sequences of ITS, ETS, and nepGS were initially aligned using MAFFT (Version 7.480, Tokyo, Japan) [21] and manually adjusted using BioEdit (Version 7.5.5, Wooster, OH, USA) [22].

Maximum-likelihood (ML) tree searches and bootstrapping (BS) were conducted for each nuclear marker using RAxML-HPC2 on XSEDE (Version 8.2.10) via the CIPRES web server [23], with 1000 bootstrap replicates. Rapid bootstrap analysis was performed with 5000 replicates, followed by a search for the best-scoring tree in a single run. Comparisons of tree topologies from the ML analyses of each individual marker did not reveal any well-supported conflicts; therefore, the three nuclear sequences were concatenated and analyzed together. Bayesian inference (BI) was conducted using MrBayes (Version 3.2.7a) [24] on the CIPRES web server, with the temperature parameter set to 0.2 and other priors left at default values. Two independent runs were performed, each with four simultaneous chains (one cold and three heated), starting from a random tree and sampling one tree every 1000 generations over 10,000,000 generations. After discarding the first 25% of trees as burn-in, the remaining trees were used to construct a majority-rule consensus tree with Bayesian posterior probabilities (PPs). ModelFinder was used to select the best-fitting substitution models for both ML and BI analyses [25], with model selection based on the Akaike information criterion (AIC) rather than the hierarchical likelihood ratio test [17].

## 5. Conclusions

Based on integrated morphological and molecular evidence, we describe *Ilex lanceifolia* K.W. Xu & Lei Jiang as a new species from Guangdong, China. The new species belongs to *Ilex.* sect. *Ilex* and is morphologically similar to *I. xiaojinensis* and *I. peiradena*, but differs in leaf blade size, petiole length, leaf length/width ratio, and the presence of prominently raised abaxial leaf veins forming distinct reticulate areoles. Phylogenetic analyses using nuclear ITS, ETS, and nepGS sequences place *I. lanceifolia* as a weakly supported sister to *I. graciliflora*, rather than with its morphological look-alikes, highlighting the complexity of morphological evolution within *Ilex*. The new species is known only from a single population in Xikeng Forest Farm, Jiangmen City, with an estimated range of less than 20 km^2^ and several thousand mature individuals; it is therefore assessed as Vulnerable (VU) under IUCN Criterion D2. The discovery of *I. lanceifolia* adds to the growing list of narrow endemic plants in the Pearl River Delta region and underscores the urgent need to conserve the remaining lowland forest fragments in this rapidly urbanizing area. Future studies employing phylogenomic approaches are needed to further resolve the relationships within *Ilex.* sect. *Ilex* and to understand the evolutionary mechanisms underlying morphological convergence in the genus.

## Data Availability

The original contributions presented in this study are included in the article. Further inquiries can be directed to the corresponding authors.

## Supplementary Materials

The following supporting information can be downloaded at https://www.mdpi.com/article/10.3390/plants15101431/s1.
